# Supplementary material for: Zero-shot medical event prediction using a generative pretrained transformer on electronic health records
Source: J Am Med Inform Assoc. 2025 Oct 8;32(12):1833–42. doi: 10.1093/jamia/ocaf160 (PMC12646381; doi:10.1093/jamia/ocaf160)
Supplement: ocaf160_Supplementary_Data [file ocaf160_supplementary_data.docx]

SUPPLEMENTARY MATERIALS

# Model Architecture and training configuration

We pretrained a causal language model on longitudinal EHR sequences using a modified GPT-2 architecture implemented with the HuggingFace Transformers library. The configuration and training setup are summarized below.

*Model Architecture.* The architecture details are provided in Table S3.

| Table S3. GPT model configuration used for EHR sequence modeling. | |
| --- | --- |
| **Parameter** | **Value** |
| Architecture | GPT2LMHeadModel |
| Hidden size | 384 |
| Number of layers | 4 |
| Attention heads | 8 |
| Context window | 513 tokens |
| Vocabulary size | 4840 |
| BOS/EOS token ID | 4839 |
| Total parameters | ∼56 million |

*Training Setup.* The model was trained on tokenized patient timelines truncated to 512 tokens per sequence.

Each input takes the form:

$$u_{i}=(V_{1}, [SEP], V_{2}, [SEP], ..., V_{L})$$

where each visit $V_{t}=(w_{t,1},w_{t,2},..., w_{t,m_{t}})$ includes diagnosis, medication, procedure, and laboratory tokens.

- **Batch size:** 256
- **Optimizer:** AdamW
- **Learning rate:** 3e-4
- **Epsilon:** 1e-8
- **Training epochs:** 2
- **Precision:** Full FP32

*Loss Function.* The model was trained using standard causal language modeling loss (next-token prediction), with attention masking applied to ignore padded positions.

*Reproducibility.* Model checkpoints and training losses were saved per epoch. The data was split at the patient level to ensure no leakage between training, validation, and test sets.

# Error analysis

To further explore the behavior of the pretrained GPT model, we visualized patient-level embeddings using UMAP for several diagnostic conditions. For each condition, we extracted the final-layer embeddings generated by the model for individual patients and projected them into a two-dimensional space using UMAP. We then labeled each point as a TP or FP based on the model’s zero-shot prediction outcome. As shown in Figure S1, there is substantial overlap between TP and FP patient representations across multiple diseases. This overlap suggests that the model assigns high confidence to patients who are, in fact, clinically similar to true positives, but ultimately do not receive the diagnosis. These errors may stem from limitations in the model’s ability to distinguish subtle clinical differences or from ambiguity in the diagnostic labels themselves (e.g., due to underdiagnosis, delayed documentation, or overlapping comorbidities). Future work is needed to better understand these borderline cases and whether incorporating richer supervision signals or patient-level context could reduce such errors.
